# Association between clinical respiratory signs, lung lesions detected by thoracic ultrasonography and growth performance in pre‐weaned dairy calves

**DOI:** 10.1186/s13620-021-00187-1

**Published:** 2021-03-25

**Authors:** Inmaculada Cuevas-Gómez, Mark McGee, José María Sánchez, Edward O’Riordan, Nicky Byrne, Tara McDaneld, Bernadette Earley

**Affiliations:** 1 Teagasc, Animal & Grassland Research and Innovation Centre (AGRIC), Grange, Co. Meath Dunsany, Ireland; 2 Meat Animal Research Center, USDA, ARS, Nebraska Clay Center, USA

**Keywords:** Bovine respiratory disease, Calf, Clinical respiratory scoring, Lung consolidation, Pneumonia, Thoracic ultrasonography

## Abstract

**Background:**

Bovine respiratory disease (BRD) is the main cause of mortality among 1-to-5 month old calves in Ireland, accounting for approximately one-third of deaths. Despite widespread use of clinical respiratory signs for diagnosing BRD, lung lesions are detected, using thoracic ultrasonography (TUS) or following post-mortem, in calves showing no clinical signs. This highlights the limitation of clinical respiratory signs as a method of detecting sub-clinical BRD. Using 53 purchased artificially-reared male dairy calves, the objectives of this study were to: (i) characterise the BRD incidence detected by clinical respiratory signs and/or TUS, (ii) investigate the association between clinical respiratory signs and lung lesions detected by TUS, and (iii) assess the effect of BRD on pre-weaning growth.

**Results:**

Clinical BRD (based on Wisconsin clinical respiratory score and/or rectal temperature > 39.6 ºC) was detected in 43 % and sonographic changes (lung lesions) were detected in 64 % of calves from purchase (23 (SD; 6.2) days of age) until weaning, 53 days post-arrival. Calves with clinical BRD were treated. Sixty-one per cent calves affected with clinical BRD had lung lesions 10.5 days (median) before detection of clinical signs. Moderate correlations (*r*_*sp*_ 0.70; *P* < 0.05) were found between cough and severe lung lesions on arrival day, and between rectal temperature > 39.6 ºC and lung lesions ≥ 2 cm^2^ on day 7 (*r*_sp_ 0.40; *P* < 0.05) post-arrival. Mean average daily live weight gain (ADG) of calves from purchase to weaning was 0.75 (SD; 0.10) kg; calves with or without clinical BRD did not differ in ADG (*P* > 0.05), whereas ADG of those with severe lung lesions (lung lobe completely consolidated or pulmonary emphysema) was 0.12 kg/d less (*P* < 0.05) than calves without lung lesions.

**Conclusions:**

Thoracic ultrasonography detected lung consolidation in calves that did not show signs of respiratory disease. The presence of severe lung lesions was associated with reduced pre-weaning growth. These findings emphasise the importance of using TUS in addition to clinical respiratory scoring of calves for an early and accurate detection of clinical and sub-clinical BRD.

## Background

Bovine respiratory disease (BRD) is a major health challenge for the cattle industry, and particularly young calves [1, 2]. It is a significant cause of morbidity and mortality in pre-weaned and weaned calves [1, 2], accounting for 34.3 per cent of deaths in calves between 1 and 5 months-old in Ireland [3]. Bovine respiratory disease, including both clinical and sub-clinical, in calves causes substantial economic losses evidently associated with veterinary costs and mortality [4], but also less apparent costs associated with reduced average daily live weight gain (ADG), carcass weight [5, 6] and reduced milk production in female dairy heifers [7].

The evaluation of clinical respiratory signs, whether through clinical respiratory score (CRS) charts, auscultation [8, 9] or subjective criteria (e.g. depressive attitude, appearance) [10], is the main approach used for diagnosing BRD and establishing criteria for treatment. However, these methods are limited since they fail to detect calves with lung lesions but not showing clinical respiratory signs (sub-clinical BRD) [10, 11]. In North America, ultrasonographic examinations of calves revealed a high prevalence of lung lesions (64 %) compared to calves diagnosed with BRD using CRS (26 %) [11]. Therefore, in order to detect these sub-clinical BRD cases, evaluation of lung appearance on the live animal is required, and this is performed using thoracic ultrasonography (TUS). The TUS instrument enables a rapid (1 min/calf), non-invasive and on-site detection of lung lesions in the live animal [12]. Despite the fact that neither CRS or TUS are gold standard methods to asses BRD status of calves, previous authors reported a greater sensitivity (62 % vs. 79 %) and specificity (74 % vs. 94 %) of TUS compared with CRS to diagnose BRD in pre-weaned dairy calves [13]. There are a limited number of studies evaluating the association between growth performance and TUS findings, in pre-weaned calves [11, 14].

Consequently, the objectives of the present study were to, (i) characterise the BRD incidence detected by clinical respiratory signs and/or TUS, (ii) investigate the association between clinical respiratory signs and lung lesions detected by TUS, and (iii) assess the effect of BRD on pre-weaning growth performance of purchased artificially-reared male dairy calves.

## Results

### Incidence of BRD and disease evolution

The classification of calves with clinical BRD (cBRD) and TUS score (TUSS) 1 to 4 from day (d) 0 to d 14 (TUS14) and d 30 (TUS30) post-arrival is represented in Table 1.

**Table 1:** Distribution of calves according to clinical bovine respiratory disease (cBRD) and thoracic ultrasonography score

TUSS^a^	TUS14^b^			TUS30^c^		
	**cBRD-**^**d**^	**cBRD +** ^**e**^	TOTAL	**cBRD-**^**d**^	**cBRD +** ^**e**^	TOTAL
**TUSS1**	22	6	28	15	4	19
**TUSS2**	3	8	11	9	8	17
**TUSS3**	2	5	7	3	6	9
**TUSS4**	3	4	7	3	5	8
**Sum of TUSS 1 to 4**	30	23	53	30	23	53

^a^Thoracic ultrasonography score (from 1 to 4) (TUSS1 (normal lung or comet tails); TUSS2 (Lung lesion < 2cm^2^; TUSS3 Lung lesions ≥ 2cm^2^; TUSS4 Consolidated lung lobe and or/emphysema)

^b^Classification of calves considering the ultrasound evaluations performed from arrival to day 14

^c^Classification of calves considering the ultrasound evaluations performed from arrival to day 30

^d^cBRD- calves that had no BRD treatment from arrival until weaning

^e^cBRD+ calves that had BRD treatment from arrival until weaning

**Fig. 1:**
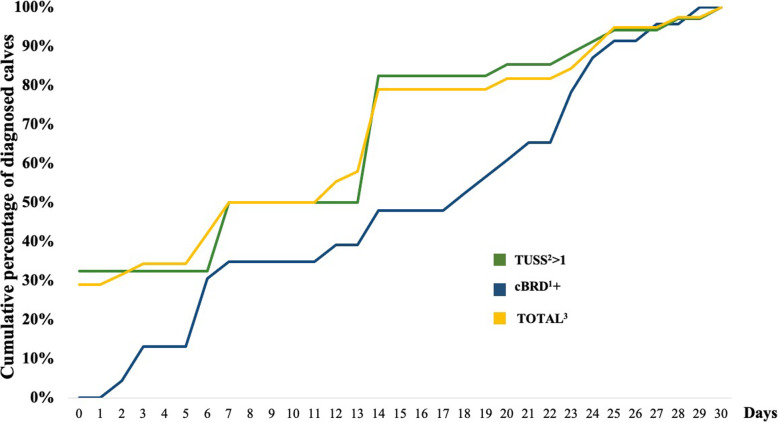
Cumulative percentage of calves diagnosed with clinical BRD or lung lesions during 30 days after arrival. ^1^Clinical bovine respiratory disease. ^2^Thoracic ultrasonography score. ^3^Calves with cBRD and/or TUSS > 1.

According to the cBRD classification, (23/53) 43 % of calves were categorised as cBRD + and (30/53) 57 % as cBRD- from arrival until weaning. From d 0 to d 14 post-arrival, 47 % of calves were detected to have lung lesions (TUSS2, 3 or 4). From d 0 until d 30 post-arrival, (34/53) 64 % of calves had lung lesions, of which (17/34) 50 % were classified as TUSS2, (9/34) 26 % as TUSS3 and (8/34) 24 % as TUSS4. Of the 34 calves with lung lesions, 19 were clinically detected and thereby treated, whereas 15 were not detected using clinical respiratory signs. Of the 23 cBRD + calves, 14 had lung lesions present a median of 10.5 days (range from 3 to 29) before detection of clinical signs. The cumulative percentage of diagnosed cBRD + calves and those TUSS > 1 during 30 d after arrival is represented in Fig. 1. By d 14, 79 % of the calves (30/38) with clinical or sub-clinical BRD had been detected. No calves developed cBRD after d 30 until weaning.

Descriptive statistics for clinical respiratory signs and presence of lung lesions on d 0, 7 and 14 are shown in Table 2.

**Table 2:** Frequencies of clinical respiratory signs and lung lesions on days 0, 7 and 14

	Day 0		Day 7		Day 14	
	**n**	**%**	**n**	**%**	**n**	**%**
**Clinical signs**^**a**^:						
RT^b^ > 39.6 °C	2	3.8	3	5.7	1	1.9
Cough	1	1.9	7	13.2	7	13.2
Nasal discharge	0	0	16	30.2	20	37.7
Eye discharge	0	0	7	13.2	8	15.1
Ear drooping	0	0	2	3.8	1	1.9
**TUSS**^**c**^:						
2	5	9.4	2	3.8	10	18.9
3	4	7.6	6	11.3	5	9.4
4	2	3.8	6	11.3	5	9.4

^a^Calves showing each clinical respiratory sign associated with bovine respiratory disease.

^b^Rectal temperature (RT).

^c^Thoracic ultrasonography score (TUSS). For more details see material and methods section.

No clinical signs were detected in calves on d 0 except for (2/53) 3.8 % and (1/53) 1.9 % of calves that showed rectal temperature > 39.6 ºC and cough, respectively; however, (11/53) 20.8 % of calves showed sonographic changes on d 0. On d 7 and 14 post-arrival, the most frequent clinical sign was nasal discharge ((16/53 calves) 30.2 % and (20/53 calves) 37.7 %, respectively), whereas there was a relatively low frequency (from (1/53 calves) 1.9 % to (8/53 calves) 15.1 %) for the remaining clinical signs. Lung lesions were detected in (14/53) 26.4 % and (20/53) 37.7 % of calves on d 7 and 14, respectively.

### Association between clinical respiratory signs and lung lesions

The association between clinical respiratory signs and TUSS is presented in Table 3. A moderate positive correlation (*r*_sp_ 0.70; *P* < 0.05) was found between presence of cough and TUSS4 on d 0. Moreover, there was a moderate positive correlation (*r*_sp_ 0.43; *P* < 0.05) between rectal temperature > 39.6 ºC and TUSS3 on d 7.

**Table 3:** Spearman rank correlations between clinical respiratory signs and lung lesions detected by thoracic ultrasonography

Clinical signs^a^	Day 0			Day 7			Day 14		
	**TUSS2**^**b**^	**TUSS3**^**b**^	**TUSS4**^**b**^	**TUSS2**^**b**^	**TUSS3**^**b**^	**TUSS4**^**b**^	**TUSS2**^**b**^	**TUSS3**^**b**^	**TUSS4**^**b**^
RT^c^ > 39.6 °C	-0.06	-0.06	-0.04	-0.05	0.43^*^	-0.09	-0.07	0.43	-0.04
Cough	-0.04	-0.04	0.70^*^	-0.08	0.39	0.39	-0.20	0.25	0.25
Nasal discharge	-	-	-	0.09	0.15	0.02	-0.09	0.01	-0.13
Eye discharge	-	-	-	0.22	0.04	-0.14	-0.08	0.04	-0.14
Ear dropping	-	-	-	-0.04	0.24	-0.07	0.28	-0.05	-0.05

^a^Percentage of calves showing each clinical respiratory sign associated with bovine respiratory disease.

^b^Thoracic ultrasonography score (TUSS). For more details regarding TUSS see material and methods section.

^c^Rectal temperature (RT)

^*^P-value < 0.05.

### Pre‐weaning growth performance

Mean ADG of calves during the pre-weaning period was 0.75 (SD; 0.1) kg. No differences (*P* > 0.05) in ADG were observed between calves classified as cBRD + and cBRD- (Table 4). In contrast, calves classified as TUSS4 had 0.12 kg reduced ADG (*P* = 0.05) compared to calves classified as TUSS1.

**Table 4:** Multivariable linear regression model for calf pre-weaning average daily gain

Variable	Estimate average daily gain (kg)	SE	*P*-value
Intercept	0.87	0.09	< 0.001
**Clinical BRD**^**a**^			
-	Referent		
+	-0.04	0.03	0.20
**TUS30**^**b**^			
TUSS1	Referent		
TUSS2	0.02	0.03	0.44
TUSS3	-0.03	0.04	0.50
TUSS4	-0.12	0.04	0.01
**Breed**^**c**^			
HF	Referent		
HF × AA	0.04	0.05	0.50
**House**^**d**^			
FV	Referent		
NV	-0.07	0.05	0.16

^a^Bovine respiratory disease.

^b^Classification of calves considering the ultrasound evaluations performed up to day 30. For more detail regarding thoracic ultrasound score (TUSS) from 1 to 4 see details on material and method section.

^c^Breed: HF, Holstein Frisian; HF × AA, Holstein Frisian × Aberdeen Angus.

^d^House: FV, fan ventilated; NV, naturally ventilated.

## Discussion

Clinical respiratory signs are widely used to diagnose BRD in dairy calves as it can be performed on-farm by personnel other than a veterinarian [1, 8]. In this study, clinical respiratory signs were only weak-to-moderately correlated with the simultaneous presence of lung lesions, thereby failing to detect sub-clinical BRD. Moreover, calves with severe (lung lobe completely consolidated or pulmonary emphysema) lung lesions grew 0.12 kg/d less than those without lung lesions, highlighting the importance of evaluating lung appearance through thoracic ultrasonography.

The incidence of cBRD from arrival until weaning reported in this study (43 %) is intermediate to values previously reported in Irish research herds for pre-weaned dairy calves where Wisconsin CRS evaluation, or a modification of it, was used (33 % [15], 40 % [16] and 50 % [17]) A lower incidence of clinical BRD using Wisconsin CRS evaluation (13–26 %) has been reported in pre-weaned dairy calves in North America [11, 18, 19]. Variances between these studies may be explained by differences in management systems. The male calves in this study faced a number of coinciding challenges (transport, co-mingling with other animals, placement in a new housing environment and adaptation to a new diet) that may have resulted in stress, and a greater predisposition to BRD [20]; these challenges are not as common in dairy heifer rearing systems where the calves are born and remain on the birth farm as replacement heifers.

In the current study, 28 % of calves had sub-clinical BRD that were undetected using clinical respiratory signs alone. Previous authors have described variable percentages (from 23 to 67 %) of dairy calves with sub-clinical BRD in facilities in North America [11, 12]. Furthermore, in the present study, the appearance of lung lesions was detected prior to the appearance of clinical respiratory signs in (14/23) 61 % of cBRD + calves, of which 6 were TUSS2, 6 TUSS3 and 2 TUSS4. Moreover (14/34) 41 % of calves identified with lung lesions developed cBRD afterwards. Similarly, it has been reported that 64 % of calves developed clinical pneumonia subsequent to the detection of lung lesions [11]. These results suggest that TUS can detect sub-clinical BRD before the detection of clinical respiratory signs, implying that TUS should be used in addition to CRS for an early and accurate diagnosis of the different BRD forms. Antibiotic treatment should be considered only in inappetent and febrile calves.

The severity of lung lesions was reduced in 32% of cases after antibiotic treatment in the present study. Lung tissue has the capacity to repair and regenerate damaged cells after disease in humans and mice [21, 22], though there is no information in cattle. It is likely that animals with mild lung lesions in this study responded well to treatment. However, the evolution of lung lesions after therapy was not part of this study and further research is warranted to address this.

In the present study the majority (79 %) of clinical and sub-clinical cases were detected by d 14 post-arrival with all cases detected by d 30. The temporal proximity between diverse stressors such as transport, housing and dietary changes predispose calves to the development of BRD soon after these stressors [23]. In feedlots, where similar stressors to those in this study (transport, new environment and diet) affect calves, BRD incidence density was greater during the first week after arrival and subsequently decreased [24]. Similarly, in a study performed in recently-weaned 7 month old beef calves in Ireland, 50 % of BRD cases were detected by d 7 after arrival to the facilities and 80 % by d 14, with no more BRD cases detected after d 28 [25]. Therefore, intensifying the monitoring of calves through CRS and TUS during the first weeks post-arrival may facilitate the detection of the majority of BRD-affected calves.

Only two moderate correlations were found between clinical respiratory signs and TUSS3 and 4 in our study. Likewise, Leruste et al. [10] reported weak correlations (*r*_sp_ from 0.16 to 0.40) between clinical respiratory signs and moderate-to-severe lung lesions at slaughter, and no correlation with mild lung lesions. Intranasal vaccination of calves at arrival in the present study could have affected the correlations between lung lesions and clinical signs as previous authors reported a reduction of lung lesions in animals that received intranasal vaccination while the clinical signs remained unaffected [26]. Clinical signs are generally used at farm level to diagnose BRD ante-mortem and as a criteria for treatment [1, 8]; however, this methodology fails to detect calves with lung lesions that may either develop into clinical BRD or go undetected as sub-clinical cases of BRD, causing important economic losses [27]. Taking into consideration the development of resistance to antimicrobials, a recent study by our group highlighted the importance of the prudent use of antibiotics at farm level [28]. In agreement with the present study, we reported that the highest risk period for disease in artificially reared calves was between birth and 30 days of age, with approximately two-thirds of all disease events occurring during this time period [28].

It is acknowledged that employment of TUS is limited as a routine diagnostic tool, particularly in large high-throughput facilities, where a rapid, repeatable, cost-effective method for prediction of risk to develop clinical BRD or lung lesions would be necessary in order to permit early intervention strategies. Additionally, further research is also warranted on antibiotic treatments based on sensitivity testing and the identification of resistant microbiomes which would inform their prudent use.

Interestingly, calves classified as TUSS4 in the pre-weaning period had a 13.8 % lower ADG compared with those classified as TUSS1. Similarly, a previous study performed with dairy heifer calves reported a 14 % poorer ADG (0.73 vs. 0.85 kg/d, respectively) in calves with lung lesions ≥ 1 cm^2^ compared with those without lung lesions [11]. In contrast, based on the presence of clinical respiratory signs in the current study there was no difference in ADG between cBRD + and cBRD – calves. However, this lack of difference could be due to the daily examinations performed by the technical farm staff that led to detection of visual clinical signs and subsequent treatment. TUS provides an objective evaluation of the classification of lung lesions in calves with BRD, which could not be detected using CRS alone, and the severity of which will impact negatively on growth performance. Therefore, although more time is required, veterinarians should consider the combined use of CRS with TUS for a more accurate diagnosis of all forms of BRD in unweaned calves.

## Conclusions

The use of clinical respiratory signs alone failed to detect sub-clinical BRD, whereas TUS is an effective tool for that purpose. The use of TUS in addition to CRS improved diagnosis and provided early and accurate detection of different forms of BRD. Calves with severe lung lesions (lung lobe consolidation or pulmonary emphysema) had reduced growth performance compared with calves without lung lesions. It is also important to note that finding calves with lung lesions does not mean that lung consolidation should be used as a tool for treatment purpose. Therefore, TUS could be implemented for calf health monitoring to provide an accurate description of BRD. More research is warranted concerning the antibiotic treatment of the different BRD forms, within the scope of responsible use of antibiotics.

## Methods

### Animal management

A total of 53 male dairy-bred calves, comprising of 28 HF × AA (24 (SD; 7.1) days old; 53.5 (SD; 8.8) kg) and 25 HF (21 (SD; 4.6) days old; 51.7 (SD; 5.4) kg), were purchased from 13 different commercial farms during February and March 2020 and transported by road to Teagasc, Grange Research Centre (transport duration ranged from 30 min to 240 min). Following arrival (d 0), calves were randomly accommodated indoors in straw-bedded individual pens, in a naturally ventilated area (n = 32) or in a fan ventilated area (n = 21), until weaning at 75 (SD; 8.8) days of age (d 53). Each calf was then administered a sub-cutaneous dose of 1.5 mg meloxicam (Metacam, Boehringer Ingelheim) and 24 h later was vaccinated through single intranasal doses against bovine respiratory syncytial virus, parainfluenza-3-virus (Rispoval RS + PI3, Zoetis) and bovine alphaherpesvirus-1 (Rispoval IBR-Marker inactivated, Zoetis). Immediately following arrival calves received 2 L of oral electrolytes (Lectade Plus, Elanco). From d 1, calves were offered 2 L of milk replacer (23 % crude protein - Golden Maverick A, Volac, Co. Cavan, Ireland) at 12.5 % a concentration of 12.5 % solids, twice-daily by bucket and had *ad-libitum* access to calf starter pencils (crude protein 18 %; crude oil and fat 4.0 %; crude ash 7.5 % - Lakeland Dairies, Monaghan, Ireland) and fresh clean water. Calves were weaned when they had achieved at least 85 kg individual bodyweight. The mean indoor temperature during the study period was 7 °C (range from 2 to 16 °C).

### Experimental design

**Fig. 2:**
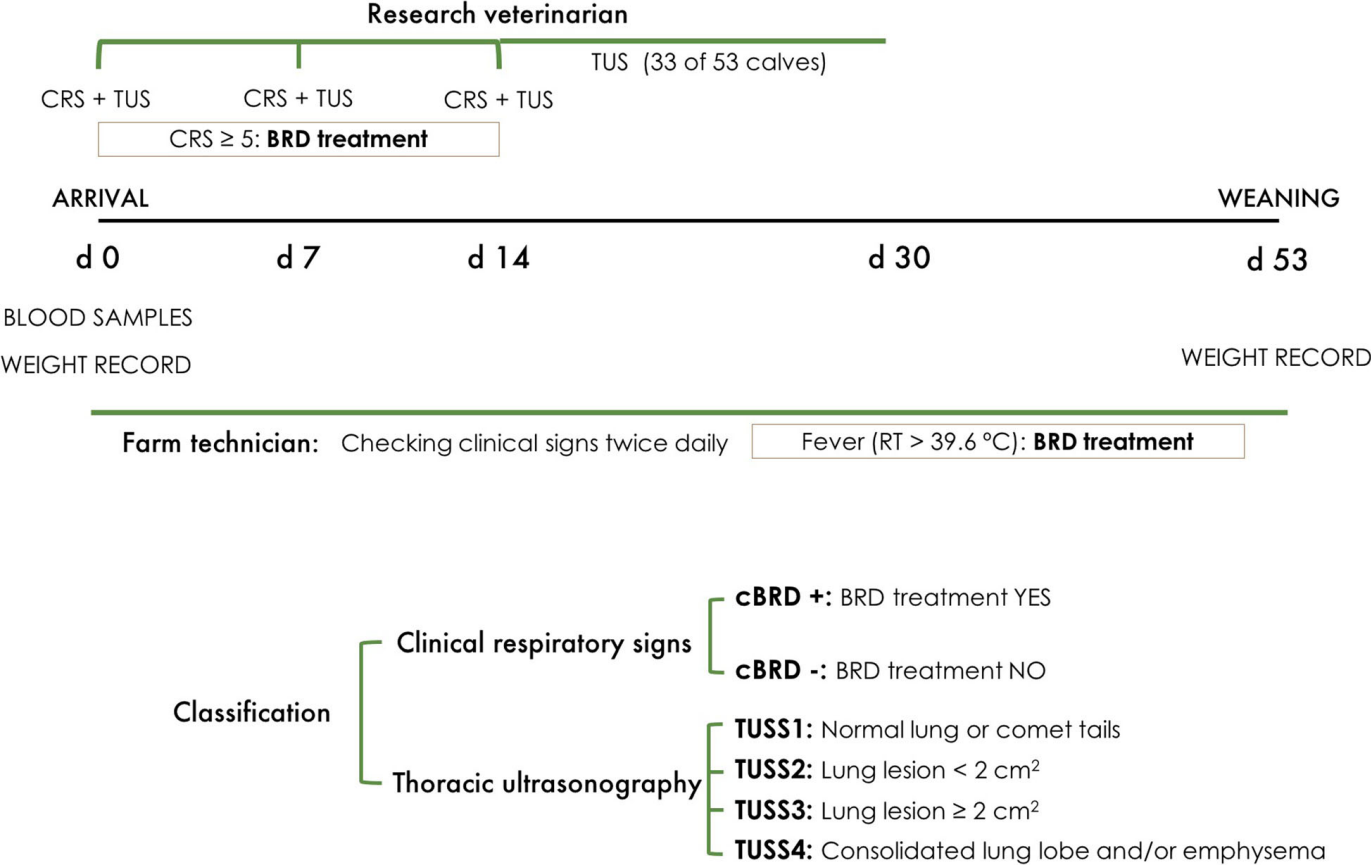
Experimental design. Timeline representing the experimental design from arrival of animals to the Research Centre facilities (d 0) until weaning (d 52 (SD; 10.4) post-arrival). Research veterinarian performed TUS and CRS on days 0, 7 and 14. Moreover, an extra TUS was performed in 33 out of 53 calves between day 14 and 30. Farm technician checked for visual clinical signs twice daily during all study period. Calves with CRS ≥ 5 and/or RT > 39.6 ºC were treated and classified as cBRD+. Animals not receiving treatment during all study period were classified as cBRD-. BRD, bovine respiratory disease; cBRD, clinical BRD; CRS, clinical respiratory score; RT, rectal temperature; TUS, thoracic ultrasonography; TUSS, thoracic ultrasonography score.

This study was a retrospective cohort study, and the experimental design is detailed in Fig. 2. The power analysis calculation was made based on analysis of data collected as part of a microbiome study, in which 20 BRD cases were necessary to detect differences compared with healthy cases. Calves were weighed using a calf weighing platform scales with Dini Argeo display (Gravitation Ltd, Ireland) at d 0 and every two weeks until weaning, d 53. All calves were observed twice daily, from arrival until weaning, for clinical signs of BRD by the same farm technician with expertise in calf health management. Recording of clinical respiratory signs (rectal temperature, cough, ear position, nasal and eye discharge) included in the scoring system of University of Wisconsin [9], and of TUS evaluations were performed by the same trained research veterinarian on d 0, 7 and 14. Thirty-three of the 53 calves, as part of calf health management, were subjected to an additional TUS evaluation between 14 and 30 d post-arrival. Rectal temperature was measured in calves showing depression, anorexia and/or dyspnoea and those with rectal temperature > 39.6 °C and/or with CRS ≥ 5 from arrival until weaning received BRD treatment consisting of a sub-cutaneous dose containing 40 mg/kg florfenicol and 2.2 mg/kg flunixin meglumine (Resflor, MSD Animal Health). Calves were classified as either cBRD+ (BRD treatment) or cBRD- (no BRD treatment) from arrival until weaning.

### Clinical respiratory score and thoracic ultrasonography

The Wisconsin CRS classifies rectal temperature, presence of cough, appearance of eye and nasal discharges, and ear position with scores ranging from 0 to 3 (from normal to very abnormal) and the sum of all scores of each clinical sign define the CRS. Calves with CRS ≥ 5 are considered as having BRD.

**Fig. 3:**
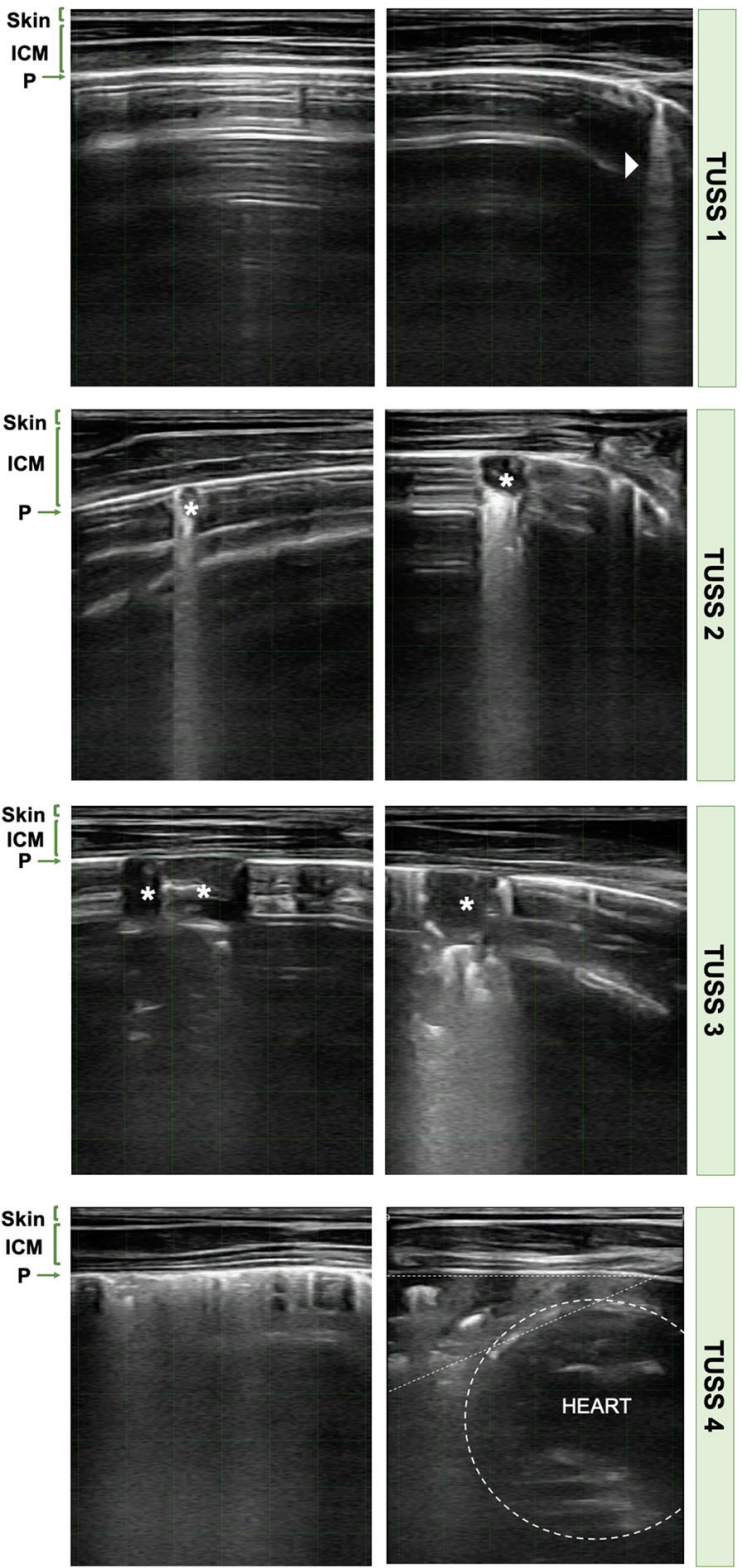
Ultrasonograms of the classifications by thoracic ultrasound score categories (8 MHz). A bright hyperechogenic line (pleura) of demarcation between the intercostal muscles and the lung tissue is observed in all ultrasonograms. The ultrasonograms of normal lung surface are classified as TUSS1, where reverberation artefacts of the pleura are observed. The presence of comet tail artefacts (arrow head) is likewise included in this category. Ultrasonograms classified in the category TUSS2 and TUSS3 include presence of lung lesions (marked with stars) with < 2 cm^2^ and ≥ 2 cm^2^, respectively. In the category TUSS4, ultrasonograms of pulmonary emphysema (to the left) or complete consolidated lung lobe (to the right) are included. The heart is delimited by circular shape dashed line and lung lobe consolidation by triangular shape dashed line. Squares delimited with green lines in the ultrasonograms images represent 1 cm^2^ each. ICM, intercostal muscles; P, pleura; TUSS, thoracic ultrasonography score.

Thoracic ultrasonography was performed as described by Ollivett et al. [12]. Briefly, an 8 MHz Wi-Fi linear transducer (Tecnoscan SR-1 C, Imporvet, Spain) was used at a maximal depth of 8 cm, and gain of 60 dB. The thorax of calves was clipped using electrical clippers and isopropyl alcohol (70 %) was used as transducing agent. Thoracic ultrasonography was performed on each side of the thorax. Systematic scanning was performed starting in the 10th intercostal space (ICS) and moving cranially until the 1st ICS to the right and the 2nd ICS to the left, sliding the transducer along the grain of the hair within each ICS. When normal lungs or presence of comet-tailing artefacts were visualised, data were annotated on-site and later transcribed to a database. In the case of lung alterations, a 10-second loop of ultrasound footage was stored to measure its size off-line after the study period was complete. For measuring consolidation size, squares in the screen representing 1 cm^2^ each, were used. Based on TUS evaluations, an adaptation of Cramer and Ollivett [11] ultrasonography score was performed to classify calves into four TUSS categories from normal lung to lung with severe lesions: TUSS1 designated those calves with normal lungs or presence of comet tails on all days when ultrasound evaluations were performed. The ultrasonogram of a normal aerated lung was characterized by the visualization of a bright hyperechoic line representing the pleura with several reverberation artefacts below it (Fig. 3, TUSS1). An ultrasonogram with one or more hyperechoic lines, also called comet tails, which perpendicularly arise from the visceral pleura indicate pleural roughening (Fig. 3, TUSS1). Calves with lung lesions < 2 cm^2^ in, at least, one assessment were considered TUSS2. Calves with lung lesions ≥ 2 cm^2^ in, at least, one evaluation were classified as TUSS3. Lung lesions (also referred to as consolidations) appear in the ultrasonogram as a hypoechoic area of varying size that disrupts the reverberation artefacts (Fig. 3, TUSS2-3). Finally, TUSS4 designated those calves with, at least, one complete consolidated lobe and/or pulmonary emphysema. A complete consolidated lobe was observed in the ultrasonogram as a wide hypoechoic area with a sonogram similar to that of the liver parenchyma. Ramifications of the fluid bronchogram were observed within the consolidated area (Fig. 3, TUSS4). The presence of emphysema was visualised in the ultrasonogram as multiple hyperechoic bands that originate from the pleura which did not permit visualisation of the lung tissue below the pleura [29] (Fig. 3, TUSS4).

### Data management and statistical analyses

Statistical analyses were performed using SAS v.9.4 software (SAS Institute Inc. Cary, NC, USA). Animal was the experimental unit. Farm of origin was included as random.

Data were checked for normality and homogeneity of variance by histograms, q–q plots, and formal statistical tests as part of the UNIVARIATE procedure of SAS. Clinical respiratory signs at d 0, 7, 14 were expressed as percentages (PROC FREQ) of observed calves that showed clinical signs (rectal temperature > 39.6 ºC, cough, nasal discharge, eye discharge and ear abnormal position) and percentage of calves with presence of lung lesions detected though TUS classified in each category. Association between presence of clinical respiratory signs and presence of lung lesions (TUSS2, TUSS3 and TUSS4) on d 0, 7 and 14 were evaluated by calculating correlation coefficients between variables using Spearman rank correlation (*r*_sp_, PROC CORR). A *post-hoc* Bonferroni correction was applied on the *P* values obtained from the multiple Spearman correlation. Univariable associations between development of cBRD or lung lesions and calf-level covariate variables (age at arrival, breed and house (natural or fan ventilated)) were tested via a *X*^*2*^ test and logistic regression analysis. Variables with a value of *P* ≤ 0.20 were included in the multivariable analyses. Calf ADG was calculated for the period from arrival until weaning. To evaluate the effect of cBRD and lung lesions had on ADG, a multivariable linear regression model (PROC GLM with MANOVA) was conducted including cBRD, TUS classification, breed and house. The Wilks’ lambda post-hoc test, multivariate version of the F-test statistic in one-way ANOVA, was applied to assesses the differences between two or more groups on multiple variables at once.

## Data Availability

All data supporting these research findings are included within the manuscript. The databases (without personally identifiable information) are available from the corresponding author upon request.

## Abbreviations

AA: Aberdeen Angus
ADG: Average daily gain
BRD: Bovine respiratory disease
cBRD: Clinical BRD
CRS: Clinical respiratory score
d: Day
HF: Holstein Friesian
TUS: Thoracic ultrasonography
TUSS: Thoracic ultrasonography score
